# Eleven years of critical obstetric pathology: epidemiologic study

**DOI:** 10.1186/cc10925

**Published:** 2012-03-20

**Authors:** L Calejman, V Nunes Velloso, E Canedo, M Deheza

**Affiliations:** 1 Hospital Rivadavia, Capital Federal, Argentina

## Introduction

The objective was to describe the characteristics of pregnant and puerperal women admitted to the ICU from February 2000 to February 2011.

## Methods

Patients admitted between the mentioned periods were grouped by age, sex, nationality, APACHE II score, days in the ICU, cause of admission: hypertensive syndromes in pregnancy (HSP), preeclampsia (P), eclampsia (E), HELLP syndrome (H), gestational diabetes, sepsis and placentary disorders; need for mechanical ventilation (MV) and dialysis, maternal mortality, and if they had proper prenatal care.

## Results

A total of 3,568 patients were admitted, from which 471 patients (13.2%) were of obstetric cause; average age was 24 years, APACHE II score of 6. There were 39 cases of arterial hypertension and 26 of diabetes mellitus before pregnancy. Sixty-eight percent were first pregnancy. The most frequent causes of admission were hypertension secondary to pregnancy (HSP) in 353 patients (75%): P 44% (*n *= 156), E 8% (*n *= 28), H 33% (*n *= 116), H/E combined 15% (*n *= 53). Other causes of admission: sepsis 16% (*n *= 75), placental disorders 7% (*n *= 33), and neurological deterioration (CVA/S SHEEHAN) 2% (*n *= 10). They required an average of two drugs to control blood pressure for the patients who needed it in 68% (*n *= 320). The average stay in the ICU was 6.5 days. From a total of 471 patients, 73 patients required mechanical ventilation (15%) and 118 (25%) patients presented high levels of urea and creatinine, 11 patients (2%) required dialysis. With respect to nationality 301 patients (64%) were Argentinean, the others reported were from Bolivia, Paraguay and Peru. Prenatal checks occurred in only 35% (*n *= 165) of the patients. The mortality rate was 6% (*n *= 28).

## Conclusion

Critical obstetrical pathology is common in the ICU, HSP as the main cause. A high number of first pregnancy patients with little prenatal care was observed. This type of patient requires low levels of life support.

